# Genome-wide mapping and analysis of aryl hydrocarbon receptor (AHR)- and aryl hydrocarbon receptor repressor (AHRR)-binding sites in human breast cancer cells

**DOI:** 10.1007/s00204-017-2022-x

**Published:** 2017-07-05

**Authors:** Sunny Y. Yang, Shaimaa Ahmed, Somisetty V. Satheesh, Jason Matthews

**Affiliations:** 1 Department of Pharmacology and Toxicology, University of Toronto, Toronto, Canada; 2 Present Address: Department of Pharmaceutical Sciences, University of British Columbia, Wesbrook Mall, Vancouver, V6T 1Z3 Canada; 3 Department of Nutrition, Institute of Basic Medical Sciences, University of Oslo, Blindern, 1046, 0317 Oslo, Norway

**Keywords:** Aryl hydrocarbon receptor (AHR), Aryl hydrocarbon receptor repressor (AHRR), 2,3,7,8-Tetrachlorodibenzo-*ρ*-dioxin (TCDD), Aryl hydrocarbon response element (AHRE), Chromatin immunoprecipitation with next-generation sequencing (ChIP-Seq), DNA binding

## Abstract

The aryl hydrocarbon receptor (AHR) mediates the toxic actions of environmental contaminants, such as 2,3,7,8-tetrachlorodibenzo-*ρ*-dioxin (TCDD), and also plays roles in vascular development, the immune response, and cell cycle regulation. The AHR repressor (AHRR) is an AHR-regulated gene and a negative regulator of AHR; however, the mechanisms of AHRR-dependent repression of AHR are unclear. In this study, we compared the genome-wide binding profiles of AHR and AHRR in MCF-7 human breast cancer cells treated for 24 h with TCDD using chromatin immunoprecipitation followed by next-generation sequencing (ChIP-Seq). We identified 3915 AHR- and 2811 AHRR-bound regions, of which 974 (35%) were common to both datasets. When these 24-h datasets were also compared with AHR-bound regions identified after 45 min of TCDD treatment, 67% (1884) of AHRR-bound regions overlapped with those of AHR. This analysis identified 994 unique AHRR-bound regions. AHRR-bound regions mapped closer to promoter regions when compared with AHR-bound regions. The AHRE was identified and overrepresented in AHR:AHRR-co-bound regions, AHR-only regions, and AHRR-only regions. Candidate unique AHR- and AHRR-bound regions were validated by ChIP–qPCR and their ability to regulate gene expression was confirmed by luciferase reporter gene assays. Overall, this study reveals that AHR and AHRR exhibit similar but also distinct genome-wide binding profiles, supporting the notion that AHRR is a context- and gene-specific repressor of AHR activity.

**Electronic supplementary material:**

The online version of this article (doi:10.1007/s00204-017-2022-x) contains supplementary material, which is available to authorized users.

## Introduction

The aryl hydrocarbon receptor (AHR) is a ligand-activated transcription factor and member of the basic helix–loop–helix (bHLH) Per-AHR nuclear translocator (ARNT)-Sim (PAS) protein family that mediates the toxic actions of environmental contaminants, such as 2,3,7,8-tetrachlorodibenzo-*ρ*-dioxin (TCDD) (Gu et al. 2000). The AHR is also involved in several other biological functions including vascular development, the immune response, and cell cycle control (Fernandez-Salguero et al. 1995; Puga et al. 2002; Schmidt et al. 1996). Unliganded AHR is sequestered in the cytoplasm by chaperone proteins including heat-shock protein 90 (Hsp90), AHR-interacting protein (AIP), and 23-kDa co-chaperone protein (p23). Upon ligand binding, AHR translocates to the nucleus and heterodimerizes with ARNT. The AHR–ARNT complex regulates transcription by binding with high affinity to specific DNA sequences termed aryl hydrocarbon response elements [AHREs; xenobiotic response elements (XREs); dioxin response elements (DREs)] located in the regulatory regions of target genes including *cytochrome P450 1A1* (*CYP1A1*), *CYP1B1*, and *TCDD*-*inducible poly*-*ADP*-*ribose polymerase* (*TIPARP*) (Hankinson 1995; Ma et al. 2001). Genome-wide analysis of AHR- and ARNT-binding sites showed overlapping profiles supporting the importance of the heterodimerization complex in DNA binding (Lo and Matthews 2012). Although AHREs are enriched in AHR-binding sites, not all of the binding sites necessarily contain an AHRE as determined in several studies (Dere et al. 2011; Lo and Matthews 2012). Similar to other ligand-activated transcription factors, AHR also binds to genomic regions 10 kb away from known promoters, suggesting a long-range regulation through a chromatin-looping or remodeling mechanism (Dere et al. 2011).

The mechanism by which AHR regulates its target genes is relatively well understood; however, our understanding of how transcriptionally activated AHR is regulated or inhibited is incomplete. Proposed mechanisms of regulation include negative feedback regulation via the aryl hydrocarbon receptor repressor (AHRR), ligand-induced proteolytic degradation via the ubiquitin/proteasome pathway, and increased metabolism of the activating ligand (Hankinson 1995; Mimura et al. 1999). AHRR is an AHR target gene and also a member of the bHLH superfamily of proteins. AHRR binds to AHREs, but lacks a ligand-binding domain (PAS B) and does not have a transactivation domain (Mimura et al. 1999). AHRR was originally described to be part of an evolutionarily conserved negative feedback loop regulating AHR activity (Evans et al. 2008; Mimura et al. 1999). AHRR heterodimerizes with ARNT and AHR, and the AHRR–ARNT complex binds to AHREs (Kikuchi et al. 2003; Mimura et al. 1999). AHRR was proposed to repress AHR by forming AHRR–ARNT complexes that subsequently compete with AHR–ARNT complexes for binding to AHREs (Mimura et al. 1999). However, overexpression of ARNT fails to rescue AHRR-dependent repression of AHR and mutation of the DNA-binding domain does not affect the ability of AHRR to repress AHR (Evans et al. 2008). This suggests that repression may occur through mechanisms that involve protein–protein interactions or that do not require ARNT. AHRR does not affect AHR protein levels, showing that the mechanism of repression is not related to increased AHR turnover (Evans et al. 2008; MacPherson et al. 2014).

AHRR has also been reported to function as a tumor suppressor in multiple types of cancer, including breast cancer (Kanno et al. 2008; Schlezinger et al. 2006). The chromosomal region containing *AHRR* is frequently deleted in several types of human cancers (Zudaire et al. 2008) and the *AHRR* promoter regions is also hypermethylated in several different tumour cell lines. AHRR knockdown was also reported to increase growth and invasiveness of human lung cancer cells as well as the anchorage-independent growth of normal non-malignant human mammary epithelial cells (Zudaire et al. 2008). Whether the tumour suppressor activity of AHRR is dependent or not on its ability to inhibit AHR is not fully understood.

To better understand the role and extent to which AHRR alters AHR action, we determined the genome-wide binding profiles of AHRR in MCF-7 human breast cancer cells using chromatin immunoprecipitation followed by next-generation sequencing (ChIP-Seq). Here we show that AHR and AHRR exhibit shared and overlapping binding to 974 regions but they also had 2127 and 994 distinct regions. Our findings revealed that, while sequences co-bound by AHR and AHRR, bound by only AHR or by only AHRR displayed high number of AHREs, AHRR-bound regions mapped much closer to the promoter regions (~1 kb from the transcription start site [TSS]) of target genes when compared with AHR-bound regions. Unique AHR-only- and AHRR-only-bound regions were also identified and validated by ChIP–qPCR and luciferase assays. Overall, this study reveals previously unidentified genomic binding preference of AHRR and provides a framework to better understand the interaction between AHR and AHRR and their potential ability to regulate transcription independently.

## Materials and methods

### Chemicals and antibodies

Dimethyl sulfoxide (DMSO) (Sigma-Aldrich) and 2,3,7,8-tetrachlorodibenzo-*ρ*-dioxin (TCDD) were purchased from Accustandard (New Haven, CT, USA). Antibodies used for ChIP-Seq include anti-AHR (H-211; Santa Cruz Biotechnology, Dallas, TX, US), anti-AHRR (HPA019614; Sigma-Aldrich, St. Louis, MO, USA), and normal rabbit immunoglobulin (sc-2027; Santa Cruz Biotechnology). Protein A agarose fast flow beads (Invitrogen, Burlington, Canada) were used for all ChIP-Seq experiments. All other reagents used were of high quality and scientific standards.

### Cell culture

MCF-7 human breast cancer cells were cultured in low-glucose (1000 mg/L) DMEM (Dulbecco’s modified Eagle’s media) supplemented with 1% (*v*/*v*) penicillin/streptomycin (P/S), either 10% (*v*/*v*) fetal bovine serum (FBS) or 5% dextran-coated charcoal (DCC)-treated FBS. COS-1 African green monkey kidney cells were cultured in low-glucose DMEM supplemented with 1% P/S and 10% FBS. Cell culture media and supplements were purchased from Sigma-Aldrich.

### Chromatin immunoprecipitation sequencing (ChIP-Seq)

MCF-7 cells were seeded at a density of 3 million cells per 10-cm dish in DMEM containing 10% FBS and 1% P/S. The following day, the medium was changed to DMEM 5% DCC-FBS and 1% P/S. Forty-eight hours later, the cells were treated for 45 min or 24 h with 10 nM TCDD and ChIP assays were performed as previously described (Lo and Matthews 2012). Immunoprecipitated DNA was purified and eluted in a final volume of 40 μL of water using Qiaquick spin columns according to the manufacturer’s instructions (Qiagen, Hilden, Germany). Ten microliters of purified immunoprecipitated DNA was used for library preparation using the MicroPlex Library Preparation Kit from Diagenode following the manufacturer’s recommendations. The amplified ChIP DNA was separated by gel electrophoresis using a 2% agarose gel. DNA fragments of 300–500 bp were excised and extracted using QIAquick Gel Extraction Kit (Qiagen). Bioanalysis was then performed on the excised band at the Center for Applied Genomics (TCAG; SickKids, Toronto, ON, Canada) using Agilent 2200 TapeStation. Three biological replicates were sequenced from DNA isolated after immunoprecipitation of AHR at 45 min, AHR at 24 h and AHRR 24 h after DMSO and TCDD treatments. Sequencing was performed at TCAG using the Illumina HiSeq 2500 sequencer.

### Identification of binding regions and overlaps

The Illumina raw output FASTQ files were mapped to the human genome assembly (hg19) using Bowtie2 (Langmead and Salzberg 2012) with output to BAM files. The data have been deposited in NCBI’s Gene Expression Omnibus (Edgar et al. 2002) and are accessible through GEO Series accession number GSE90550 (https://www.ncbi.nlm.nih.gov/geo/query/acc.cgi?acc=GSE90550). The three replicates were pooled using SAMTools (Li et al. 2009). Peak-calling was performed using Model-based Analysis for ChIP-Seq (MACS2) program using default settings (Zhang et al. 2008). To investigate the genomic binding of AHR and AHRR, we used a solo-peak-calling approach (Gualdrini et al. 2016), in which the DMSO- or TCDD-treated datasets were peak-called using an assumed even background signal. For identification or peak-calling of TCDD-induced AHR- and AHRR-bound regions, the pooled TCDD-treated BAM file was inputted as the treatment and the pooled DMSO-treated BAM file was inputted as the control in MACS2. The output BED files contained all of the peak regions that passed the *q* value cutoff of 0.05. To remove the high-risk regions (regions with high ChIP signals such as near centromeres, telomeres, satellite repeats), the ENCODE consortia blacklisted regions (Consortium EP 2012) were filtered out using BEDTools (Quinlan and Hall 2010). Integrative Genomic Viewer (IGV) was used for visualization of signal peaks (Thorvaldsdottir et al. 2013). Overlap analysis and manipulation of BED files were done using BEDTools. Overlap analysis was performed with the 24-h TCDD-induced AHR- and AHRR-bound regions as well as another dataset from a different study, the 45-min TCDD-induced AHR-bound regions. Because the 45-min TCDD-treated AHR solo-peak-called regions list resulted in the highest number of peaks for AHR, we used this dataset as a stricter filter to identify unique AHRR-bound regions.

### ChIP-seq analysis (de novo motif, gene list)

The Hypergeometric Optimization of Motif EnRichment (HOMER) Analysis Suite was used for peak annotations of genomic features (Heinz et al. 2010). The Discriminative Regular Expression Motif Elicitation (DREME) (Bailey 2011) and Sampling with Expectation Maximization for Motif Elicitation (SEME) (Zhang et al. 2013) programs were used for de novo motif discovery. The output position weighted matrix file from SEME was designed into logos and matched with JASPAR database using STAMP with default settings (Mahony and Benos 2007). Overrepresented transcription factor-binding site (TFBS) analysis was performed using Genomatix Software Suite (http://www.genomatix.de) based on the number of matches in ChIP sample compared to genomic background or promoter background for AHRR-only regions. Top canonical pathways and functions were predicted for AHR- and AHRR-bound genes with the Ingenuity Pathway Analysis (IPA) software (Ingenuity Systems, Inc., Redwood, CA, USA).

### ChIP–qPCR validation

Selected regions derived from the overlap analysis were validated by ChIP–qPCR. AHRR-unique regions were selected such that they did not overlap with or were not annotated to the same closest gene as any AHR-bound regions from both the 45-min and 24-h dataset. Similar methods were applied when validating AHR unique regions. Sequences for qPCR primers used to amplify the ChIP regions are provided in Supplementary Table S1.

### Reporter gene assay

Selected unique AHR and AHRR regions that were validated by ChIP–qPCR were then PCR amplified and cloned into the luciferase basic (pGL3-basic) or promoter (pGL3-promoter) reporter vectors (Promega, Madison, WI, USA). The selected AHRR-only-binding ChIP region annotated to *X*-*ray repair cross*-*complementing protein 6* (*XRCC6*), which was the closest TSS to the AHRR-only bound region, was located within the promoter region of the gene (sequence from the SwitchGear promoter database). To investigate whether AHRR regulates the expression of the gene with only the ChIP region, the promoter region that contained AHRR-bound ChIP region of an AHRR-only gene, *XRCC6* was cloned into the pGL3-basic plasmid. However, the selected AHR-only bound region, annotated to *cannabinoid receptor 2* (*CNR2*), was not found within the promoter region of the genes. These regions were cloned into a pGL3-promoter vector, which contains an SV40 promoter, to examine their potential enhancer activity. Primers were designed to introduce the MluI and BglII restriction enzyme sites into the PCR products. The regions of interest were PCR amplified from genomic DNA from MCF-7 cells with the following set of primers (Supplementary Table S1). Amplification of the PCR products was done using *Pfu* Turbo DNA polymerase (Agilent). These PCR products were then digested with MluI and BglII restriction enzymes. The primers used for cloning of the reporter gene constructs are provided in Supplementary Table S1. The reporter gene constructs were further validated by sequencing. For the transfection experiments, varying levels (0, 100, 200, 400 ng) of pcDNA–AHR, pcDNA–ARNT and pcDNA–AHRRΔ8 expression vectors were transfected into COS-1 cells along with 200 ng of reporter gene luciferase vectors using lipofectamine 2000 (Invitrogen) (MacPherson et al. 2014). Six hours after transfection, cells were treated for approximately 20 h with DMSO, 10 nM TCDD, and/or 100 nM TCDD. As a positive control, pGL3–CYP1B1–Luc was transfected under the same conditions (MacPherson et al. 2009). Luciferase activity was determined using the ONE-Glo luciferase system (Promega).

### Statistical analysis

Statistical analysis was performed using one-way or two-way analysis of variance (ANOVA) with Bonferroni post hoc test at a statistical significance of *P* < 0.05.

## Results

### Identification of TCDD-induced AHR- and AHRR-bound regions

**Fig. 1:**
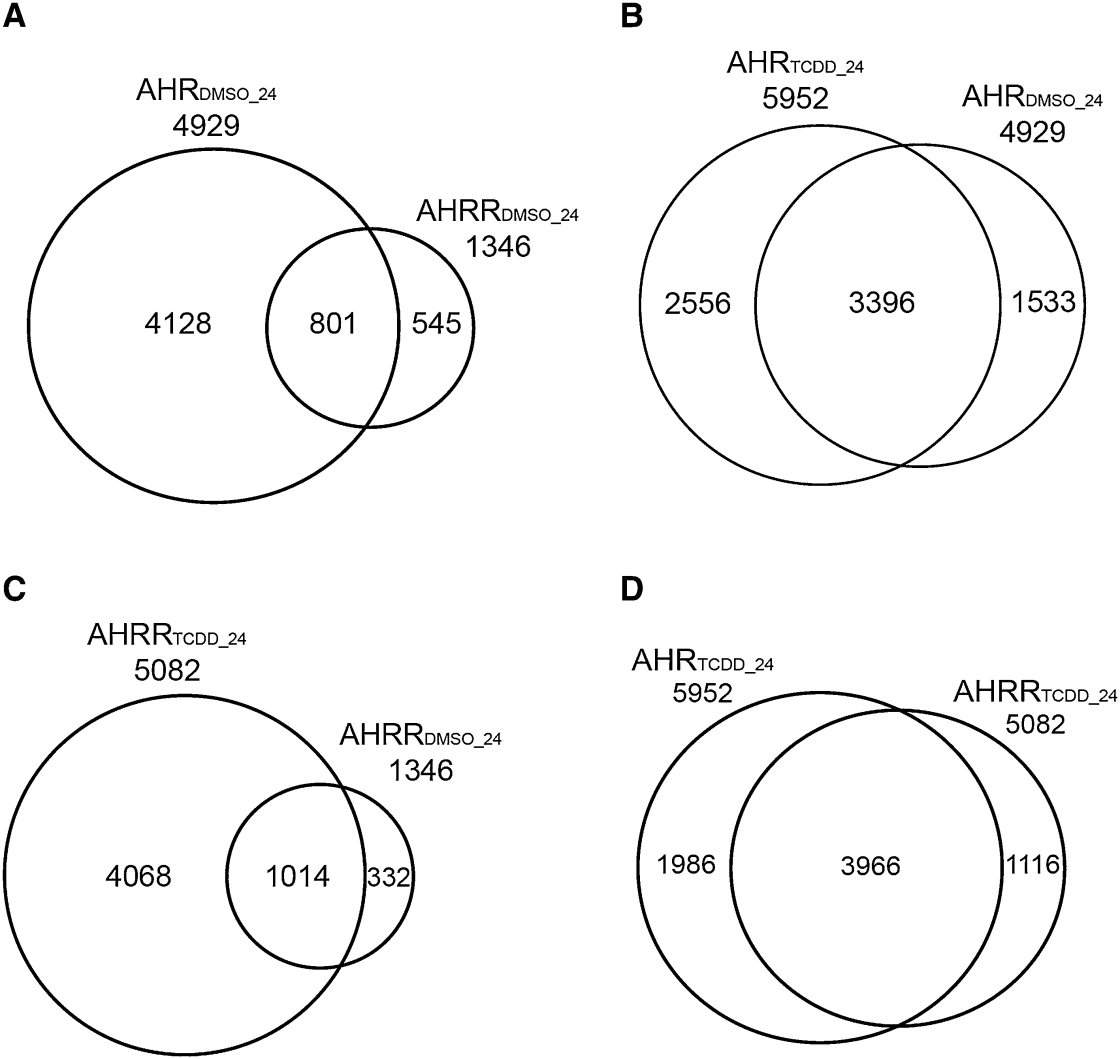
Overlap among the genomic binding sites of AHR and AHRR in the presence of DMSO or TCDD using solo-peak-calling. **a** Common genomic regions between AHR_DMSO_24_ and AHRR_DMSO_24_ after 24-h exposure to DMSO. **b** Overlap of AHR-bound regions between AHR_TCDD_24_ and AHR_DMSO_24_ after 24-h treatment with TCDD or DMSO. **c** Overlap of the AHRR-bound regions between AHRR_TCDD_24_ and AHRR_DMSO_24_ after 24-h treatment with TCDD or DMSO. **d** Overlap of the common AHR- and AHRR-bound regions after 24-h treatment with TCDD

To identify the genomic binding profiles of AHR and AHRR, we performed ChIP-Seq on chromatin isolated from MCF-7 human breast cancer cells treated with 10 nM TCDD for 24 h. The experimental conditions were selected based on our previous study where AHRR protein levels were only detected in MCF-7 cell extracts after 24-h TCDD treatment using the anti-AHRR antibody available from Sigma (HPA019614) (MacPherson et al. 2014). To determine AHR-bound regions (AHR_DMSO_24_) and AHRR-bound regions (AHRR_DMSO_24_) in the absence of a ligand, DMSO-treated samples were peak-called using a solo-peak-calling method described in Materials and Methods. As expected, there were more bound regions for AHR_DMSO_24_ than AHRR_DMSO_24_ (4929 vs. 1346) (Fig. 1a). The identification of AHRR-bound regions in the DMSO-treated samples, despite not detecting AHRR protein levels in MCF-7 cells using the anti-AHRR antibody available from Sigma (HPA019614), demonstrates the increased sensitivity of ChIP-Seq compared with western blotting (MacPherson et al. 2014). Of the 1346 AHRR_DMSO_24_, 801 of them overlapped with AHR_DMSO_24_ regions (Fig. 1a). We next determined the solo-peak-called regions for AHR (AHR_TCDD_24_) and AHRR (AHRR_TCDD_24_) after 24-h treatment with 10 nM TCDD. For AHR, TCDD treatment resulted in the identification of 5952 AHR_TCDD_ regions and 4929 AHR_DMSO_24_ regions of which 69% (3396) (Fig. 1b). For AHRR, TCDD treatment resulted in the identification of 5082 AHRR_TCDD_ regions that overlapped with 75% (1014) of the AHRR_DMSO_24_ regions (Fig. 1c). In the presence of TCDD, 78% (3966) of the AHRR_TCDD_ regions overlapped with the AHR_TCDD_ regions (Fig. 1d). These findings showed that the TCDD-dependent increase in AHRR protein levels (MacPherson et al. 2014) resulted in increased genomic binding of AHRR. The data also revealed that the binding of AHRR in MCF-7 cells represents a subset of the TCDD-induced regions. Moreover, the non-overlapped solo-peak-called regions between AHR and AHRR that were identified after DMSO or TCDD treatment were of lower statistical significance, supporting the notion that TCDD increases the binding of AHR and AHRR to regions they are bound to in the absence of TCDD. We cannot exclude the possibility that the binding of AHR and AHRR is due the endogenous or natural AHR ligands present in the serum or medium, such as tryptophan degradation products (Rannug et al. 1987).

**Fig. 2:**
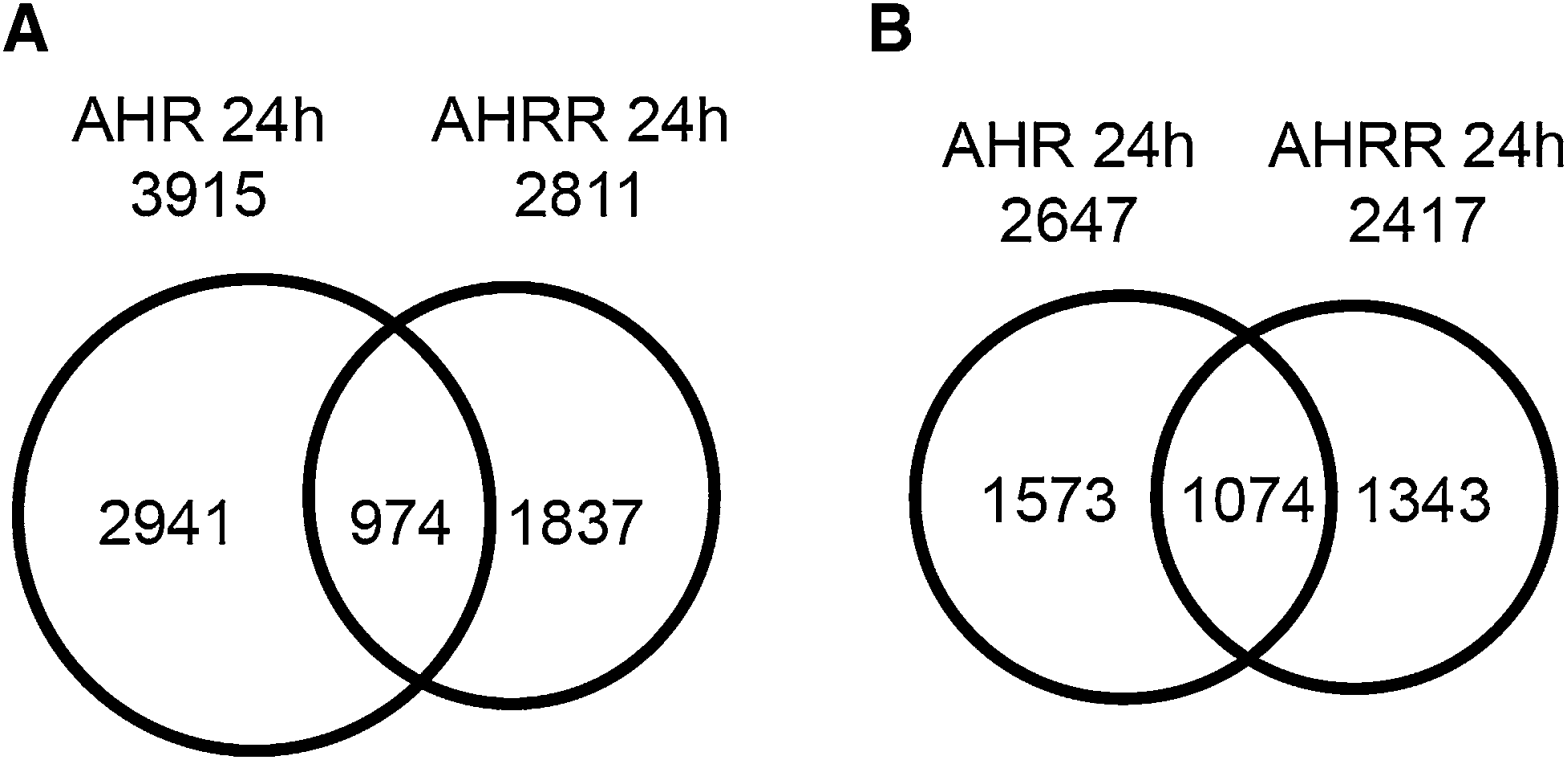
Overlap between TCDD-induced AHR_24h_ and AHRR_24h_ genomic regions (**a**) and their corresponding genes (**b**)

**Fig. 3:**
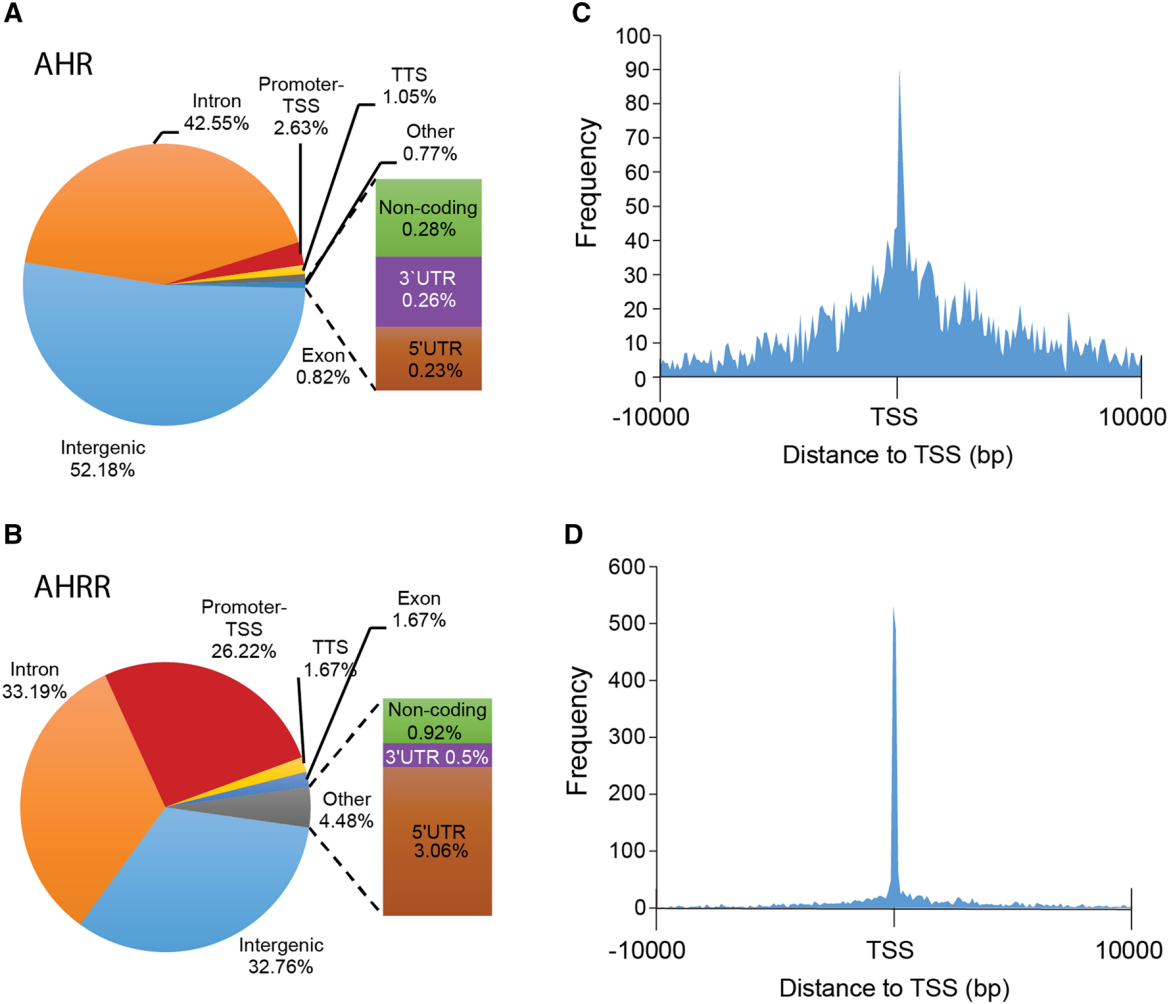
Annotated genomic locations of TCDD ligand-induced (TCDD vs. DMSO) **a** AHR- and **b** AHRR-bound regions. Histogram of the distance to the TSS for TCDD-induced **c** AHR peak regions and **d** AHRR peak regions. *TSS* transcription start site, *TTS* transcription termination site, *UTR* untranslated region

AHRR expression is induced by TCDD-dependent activation and binding of AHR to the AHRR promoter, which is part of a negative feedback loop that regulates AHR activity where AHRR is recruited to AHR-regulated genes (Mimura et al. 1999). However, AHRR has also been reported to function as a tumor suppressor independently of AHR (Kanno et al. 2008). Therefore, we reasoned that identifying AHR:AHRR-co-bound regions after TCDD treatment would support the recruitment of AHRR to AHR-regulated genes as proposed in a negative feedback loop model (Hahn et al. 2009). On the other hand, unique AHR and unique AHRR regions would provide further evidence for separate regulatory activities for both transcription factors. To this end, we treated MCF-7 cells with DMSO or 10 nM TCDD for 24 h to induce AHRR protein levels (MacPherson et al. 2014) prior to performing ChIP-Seq. We then identified TCDD-induced AHR- and AHRR-bound regions by determining regions that were significantly increased by TCDD compared with DMSO treatment for both AHR and AHRR. Using this approach, we identified 3915 TCDD-induced AHR-bound (AHR_24h_) regions and 2811 AHRR-bound (AHRR_24h_) regions using MACS2 and a *q* value cutoff of 0.05. After annotation to the closest genes, these binding regions corresponded to 2647 AHR_24h_ and 2417 AHRR_24h_ genes (Fig. 2a, b). We next used BEDTools to determine the overlap between the AHR- and AHRR-bound regions and identified 974 co-bound regions. There was a higher percentage of overlap of annotated genes compared with the overlap of binding regions suggesting that AHR and AHRR bind to different locations to regulate the same gene (Fig. 2a, b). The genomic locations of AHR_24h_ and AHRR_24h_ regions were divided into eight categories (intergenic, intron, promoter–TSS, transcription termination site [TTS], exon, non-coding, 3′-UTR [untranslated region], 5′-UTR, other) (Fig. 3a, b). The most notable differences between the AHR- and AHRR-binding locations was the high promoter–TSS binding of AHRR (26.2% in the AHRR peak regions compared with only 2.6% in the AHR regions) and 5′-UTR (3.1 vs. 0.2%) (Fig. 3a). This difference was confirmed by a peak density (number of peaks/bp) calculation that was highest immediately adjacent to TSS for AHRR_24h_ compared with AHR_24h_ regions (Fig. 3b). In agreement with previous ChIP-Seq studies, AHR had some binding preference for promoter regions with the highest peak density at the TSS (Lo and Matthews 2012); however, this promoter-centric pattern was markedly lower when compared with that of AHRR (Fig. 3c, d).

### De novo motif analysis and overrepresented transcription factor-binding site analysis

We used MatInspector to search the AHR_24h_ and AHRR_24h_ datasets for AHREs and found that 36.9% (1445) of AHR_24h_ and 64.2% (1806) of AHRR_24h_ regions contained at least one AHRE. To identify other potential binding sites in AHRR-dependent repression, we performed de novo motif discovery on the top 500 TCDD-induced AHR_24h_ and AHRR_24h_ regions, using the DREME (Table 1) and SEME algorithms (Table 2). For both AHR_24h_ and AHRR_24h_ datasets, the full pentanucleotide AHRE core consensus sequence (5′-GCGTG-3′) was the highest ranked using the SEME analysis while the quadranucleotide invariant core (5′-CGTG-3′) was the highest ranked using the DREME analysis. Other common motifs included forkhead box (FOX) and specificity protein 1 (SP1), GATA-binding proteins and activator protein-1 (AP-1) motifs were found to be unique for the AHR_24h_ dataset. As expected, an AHRE was among the top five overrepresented TFBSs for both AHR_24h_ and AHRR_24h_; however, there were some notable differences between the datasets (Table 3). GC-rich binding motifs were highly overrepresented in the AHRR dataset, which may reflect the promoter-centric binding preference of AHRR. Pathway analysis was done on the top 500 AHR_24h_ and AHRR_24h_ genes. The AHR signaling pathway was the top pathway for both datasets though it was statistically more significant for the AHR dataset (Table 4). Interestingly, the xenobiotic metabolism pathway, a known pathway activated by AHR, was not predicted for the AHRR dataset.

**Table 1:**
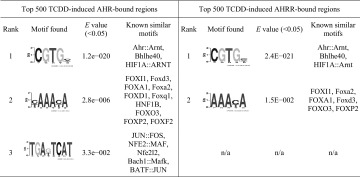
DREME motif discovery for top 500 AHR- and AHRR-bound regions (*E* value <0.05) (Bailey 2011)

A quadranucleotide version of AHRE invariant core (5′-CGTG-3′) was ranked first for both AHR and AHRR. Other motifs of interest include FOX and AP-1 (JUN and FOS) motifs for AHR

**Table 2:**
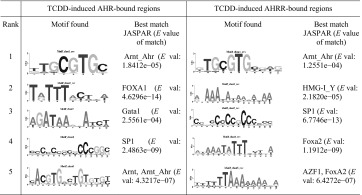
SEME motif discovery of top 5 motifs for top 500 AHR- and AHRR-bound regions with the best match in JASPAR motif database and corresponding *E* value of matching

The full AHRE core consensus sequence (5′-GCGTG-3′) was ranked first both the AHR and AHRR dataset. Other motifs found for AHR include FOXA1, GATA1, SP1, and ARNT. Both SP1 and FOX motifs were also found for AHRR

**Table 3:** Top 5 overrepresented transcription factor-binding sites (Genomatix, http://www.genomatix.de/) for top 500 TCDD-induced AHR- and AHRR-bound regions

Rank	Top 500 TCDD-induced AHR-bound regions			Top 500 TCDD-induced AHRR-bound regions		
	TF families	Description	*Z* score (genome)	TF families	Description	*Z* score (genome)
1	AHRE	AHR–ARNT heterodimers and AHR-related factors	34.2	NRF1	Nuclear respiratory factor 1	94.86
2	AP2	Activator protein 2	24.19	ZF5	ZF5 POZ domain zinc finger	88.48
3	AP1R	MAF and AP1-related factors	15.15	AHRE	AHR–ARNT heterodimers and AHR-related factors	67.65
4	AP1	AP1, Activating protein 1	15.15	EGR	EGR/nerve growth factor-induced protein C and related factors	58.42
5	ERE	Estrogen response elements	12.34	E2F	E2F-myc activator/cell cycle regulator	50.21

The AHRE was ranked first for AHR but third for AHRR. Despite a high z score for AHRE, other GC-rich transcription factor-binding sites were overrepresented in the AHRR dataset with similar or higher significance include nuclear respiratory factor 1 (NRF1), ZF5 POZ domain zinc finger (ZF5), early growth response (EGR), and E2F

**Table 4:** Top 5 canonical pathways predicted for top 500 TCDD-induced AHR-bound and AHRR-bound genes with their corresponding *P* values as calculated by the Ingenuity Pathway Analysis (IPA)

Rank	Top 500 AHR-bound genes		Top 500 AHRR-bound genes	
	Top canonical pathways	*P* value	Top canonical pathways	*P* value
1	Aryl hydrocarbon receptor signaling	1.32E−07	Aryl hydrocarbon receptor signaling	1.52E−04
2	ERK/MAPK signaling	1.37E−04	Molecular mechanisms of cancer	2.75E−04
3	Xenobiotic metabolism signaling	3.01E−04	ERK/MAPK signaling	2.21E−03
4	HGF signaling	3.06E−04	EIF2 signaling	3.91E−03
5	Molecular mechanisms of cancer	5.20E−04	Telomerase signaling	4.46E−03

Although AHR signaling pathway was ranked first for both AHR and AHRR datasets, it was predicted more significantly in the AHR dataset

### Overlap and identification of AHR- and AHRR-unique binding regions

**Fig. 4:**
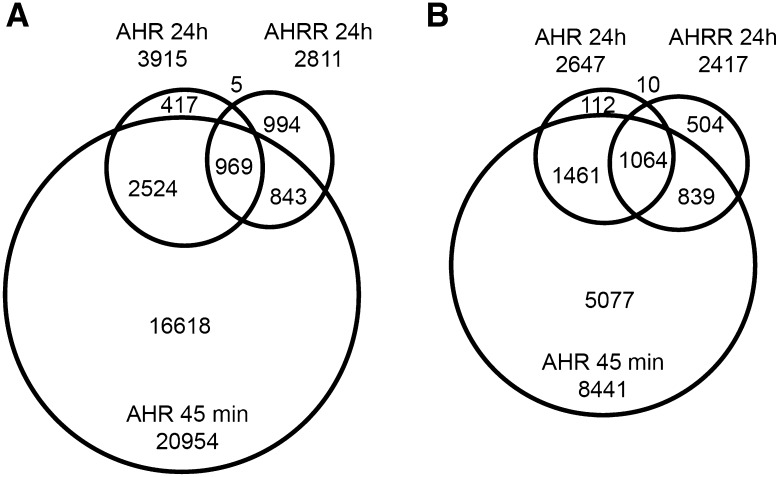
Overlap between TCDD-induced AHR_24h_, AHRR_24h_, and AHR_45min_ genomic regions (**a**) and their corresponding genes (**b**)

We previously reported that maximum AHR recruitment occurs after approximately 45 min of TCDD exposure in MCF-7 cells cultured under the conditions used in the present study and that AHR-bound regions determined at a later time points, such as 24 h, represent a subset of the AHR-bound regions present after 45 min (Dere et al. 2011; Lo and Matthews 2012) (Fig. 4). To determine high confidence unique AHRR-bound regions and to exclude the possibility that AHRR_24h_ regions overlapped with or simply represented a subset of AHR_45min_ regions, we performed ChIP-Seq for AHR-bound regions using chromatin isolated from MCF-7 cells treated with 10 nM TCDD for 45 min (AHR_45min_) and overlapped these regions with AHR_24h_ and AHRR_24h_ datasets. We identified 20954 AHR_45min_ regions using the same peak calling strategy used for the 24-h datasets. The AHR_45min_ regions corresponded to 8441 AHR_45min_ genes. We next used BEDTools to determine the overlap among all three datasets which revealed that 3493 (89%) AHR_24h_ regions or 2525 (95%) AHR_24h_ genes overlapped with AHR_45min_ regions and AHR_45min_ genes, respectively. These data support our previous findings that the AHR_24h_ represent a subset of the AHR_45min_ regions (Dere et al. 2011; Lo et al. 2011) (Fig. 4). We found a total of 1857 regions (66%) or 1913 genes (79%) that were common among AHRR_24h_ with either AHR_45min_ or AHR_24h_ datasets. We identified 994 unique AHRR_24h_ regions (AHRR-only) or 504 genes, and 2941 unique AHR regions or 1528 genes (common to both AHR_45min_ and AHR_24h_). For the unique AHR-bound regions (AHR-only), we considered the 2524 regions that overlapped between AHR_45min_ and AHR_24h_ datasets. The co-bound regions were also located close to the TSS of annotated genes but also at distant genomic sequences. The unique AHR-bound regions displayed a more dispersed binding pattern compared with any other subset of regions in our analyses. AHRR-only regions were almost exclusively located at promoters where the majority of the regions fell within 2000 bp of the TSS.

**Fig. 5:**
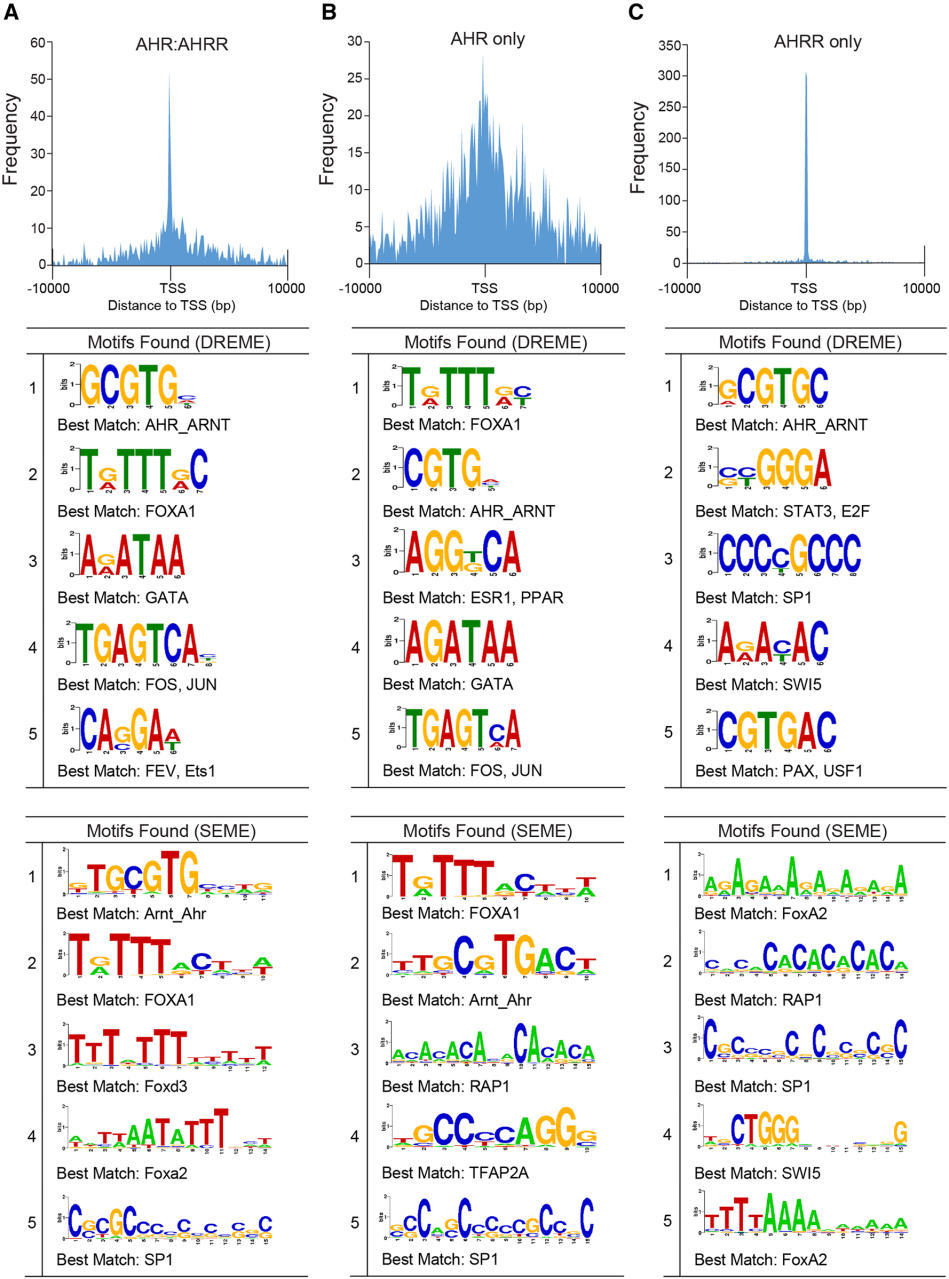
Genomic distribution and de novo motif analysis of overlapping and unique AHR and AHRR genomic regions. Histogram of the distance to TSS for TCDD-induced and top five de novo motif discovery using DREME and SEME for **a** AHR:AHRR-co-bound, **b** AHR-only and **c** AHRR-only genomic regions. *TSS* transcription start site

We next applied the same de novo motif discovery (Fig. 5) and overrepresented TFBS analysis (Table 5) analyses to the AHR:AHRR-co-bound, AHR-only and AHRR-only regions as we used on the AHR_24h_ and AHRR_24h_ datasets. As expected de novo motif discovery using DREME or SEME identified an AHRE-like motif as the top-ranked motif for the AHR:AHRR-co-bound and the second ranked for the AHR-only regions. Other notable observations for AHR:AHRR-co-bound and AHR-only regions included the high-ranked FOXA1 motif and a predicted ERE-like sequence for the AHR-only regions, the latter was only identified using DREME. Overrepresented motif analysis supported the importance of the AHREs motif and other GC-rich motifs. An ERE was also overrepresented in the AHR-only regions, but FOXA1 motifs were not highly overrepresented (Table 5). An AHRE-like motif was the top-ranked motif by DREME for AHRR-only regions; however, a similar motif was not detected using SEME (Fig. 5). Other notable transcription factor-binding motifs included FoxA2 sequences and GC-rich sequences recognized by SP1, E2F, and EGR (Table 5). For the AHRR-only regions, the AHRE was the top-ranked motif in the overrepresented transcription factor-binding site analysis. Other notable transcription factors were those that recognize GC-rich sequences including NRF1, ZF5, E2F, and EGR (Table 5).

**Table 5:** Top 5 overrepresented transcription factor-binding sites from AHR:AHRR-co-bound, AHR-only and AHRR-only regions

Overrepresentation analysis AHR:AHRR-co-bound regions				Overrepresentation analysis AHR-only			Overrepresentation analysis AHRR-only		
Rank	TF Family	Description	*Z* score	TF family	Description	*Z* score	TF family	Description	*Z* score
1	AHRE	AHR–ARNT heterodimers	42.1	AP1	Activating protein 1	17.2	AHRE	AHR–ARNT heterodimers	39.3
2	AP2	Activator protein 2	28.7	AP2	Activating protein 2	16.4	NRF1	Nuclear respiratory factor 1	18.2
3	PAX9	PAX-9 binding sites	14. 6	AP1R	MAF and AP1-related factors	14.9	ZF5F	ZF5 POZ domain zinc finger	14.8
4	AP1R	MAF and AP1-related factors	12.5	ERE	Estrogen response elements	12.0	EGRF	EGR/nerve growth factor-induced protein C	14.0
5	CTCF	CTCF and BORIS gene family	12.1	AHRE	AHR–ARNT heterodimers	10.3	E2F	E2F activator factor 1	13.8

AHR:AHRR-co-bound and AHR-only regions were probed against a genomic background, while AHRR-only were probed against a promoter background using Genomatix (http://www.genomatix.de/)

### Validation of unique AHR- and AHRR-bound genomic regions

**Fig. 6:**
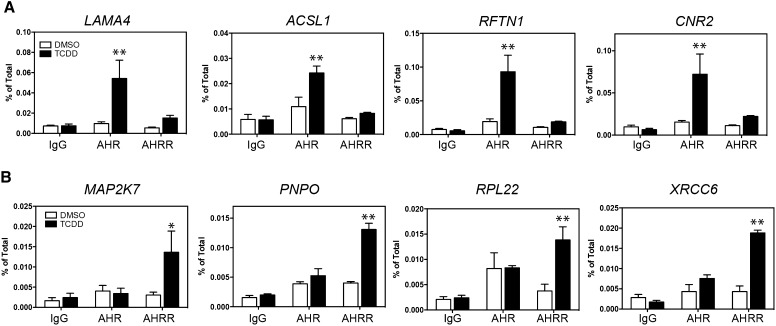
ChIP–qPCR of **a** AHR-only regions and **b** AHRR-only regions selected from ChIP-Seq analysis. The data represent three independent experiments. *Asterisks* indicate statistically significant differences compared with the antibody-matched DMSO sample (*P < 0.05, **P < 0.01) using a two-way ANOVA followed by a Bonferroni post test

We confirmed by qPCR the recruitment levels of AHR and AHRR to a subset of unique AHR-only and AHRR-only regions (Fig. 6a, b). For AHR-only regions, the chosen regions were annotated to the closest genes, *Acyl*-*CoA Synthetase Long*-*Chain Family Member 1* (*ACSL1*), *CNR2*, *laminin subunit 4* (*LAMA4*), and *raftlin, lipid raft linker 1* (*RFTN1*) (Fig. 6a). An AHRE core sequence was only identified in the *LAMA4*-bound region, whereas ACSL1, RFTN1, and CNR2 contained an AHRE-like motif (Table 6). Because AHR also binds to genomic regions 10 kb away from known promoters, other AHRE sequences through a chromatin-looping or remodeling mechanisms could influence the binding of AHR to ACSL1, RFTN1 or CNR2 (Dere et al. 2011).

**Table 6:** Comparison of the putative AHRE sequences in the AHR- and AHRR-only selected ChIP-Seq regions. Predicted AHREs were determined using MatInspector (http://www.genomatix.de/)

	Annotated gene	# of AHREs	
		[T/A/C]CGTG	GCGTG
AHR-only	*LAMA4*	0	1
	*ACSL1*	2	0
	*RFTN1*	2	0
	*CNR2*	2	0
AHRR-only	*MAP2K7*	1	3
	*PNPO*	3	1
	*RPL22*	1	3
	*XRCC6*	1	3

For AHRR-only regions, the chosen regions were annotated to the closest genes, *Mitogen*-*Activated Protein Kinase Kinase* 7 (*MAP2K7*), *pyridoxamine 5′*-*phosphate oxidase* (*PNPO*), *ribosomal protein L22* (*RPL22*), and *XRCC6* (Fig. 6b). All four of the AHRR-only regions contained at least one AHRE core (GCGTG) and one AHRE-like motif (Table 6). Using ChIP–qPCR, we confirmed the TCDD-dependent recruitment of AHR (but not AHRR), for the unique AHR-bound regions, as well as the recruitment of AHRR (but not AHR), for unique AHRR-bound regions. These results provided further validation for the unique regions identified from the ChIP-Seq analysis.

**Fig. 7:**
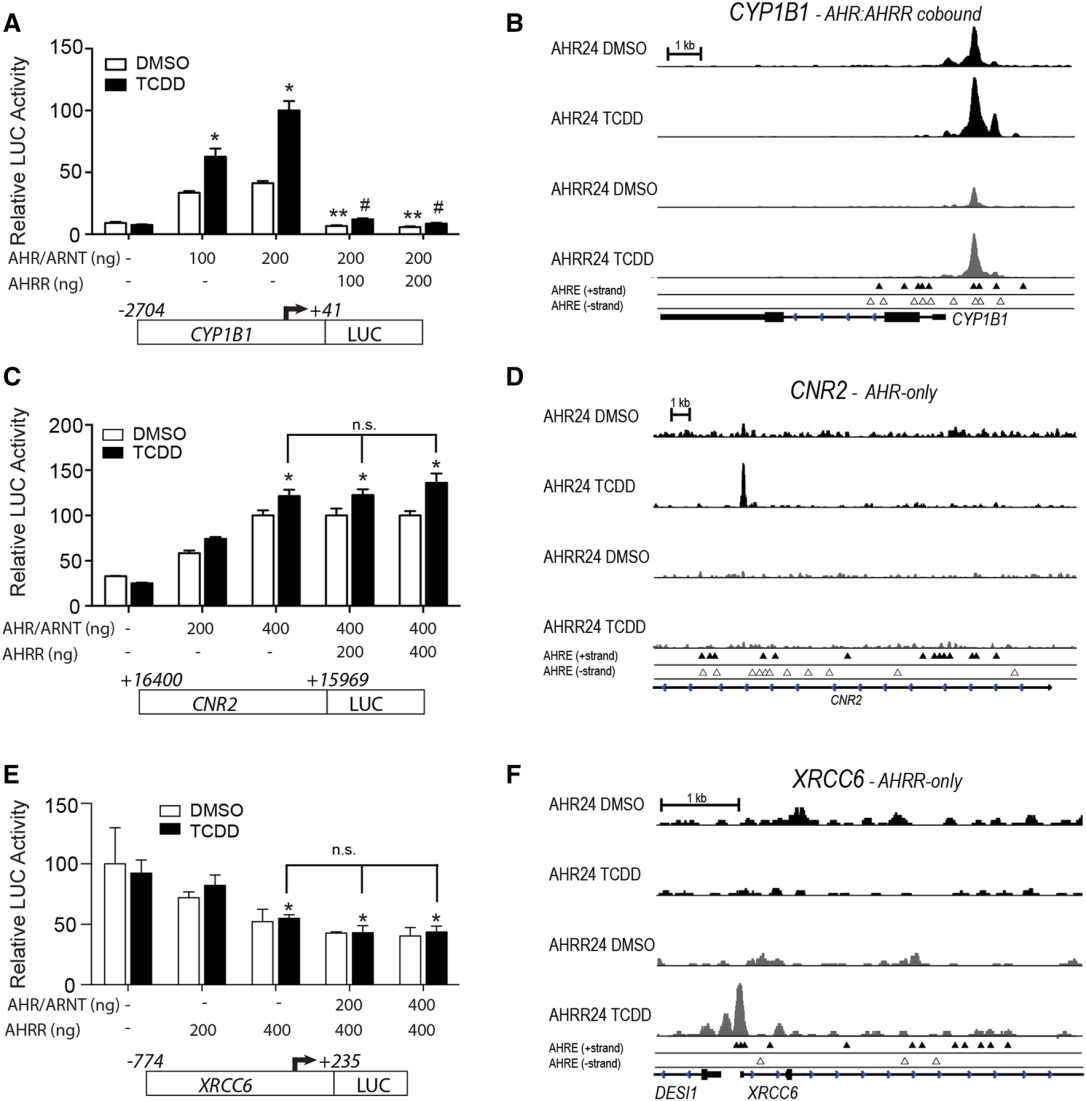
Overexpression of AHRR does not repress AHRR-regulated CNR2, while overexpression of AHR does not prevent AHR repression of XRCC6 reporter gene activity. COS1 cells were transfected with **a** CYP1B1–luciferase, **b** CNR2–luciferase or **c** XRCC6–luciferase with increasing concentrations of AHR or AHRR and then treated with 10 nM TCDD or DMSO control for 24 h. Integrative genome viewer (IGV) visualization of the AHR- and AHRR-bound peaks in the absence (DMSO) and presence of TCDD for 24 h for **b** *CYP1B1*, **d** *CNR2*, and **f** *XRCC6*. The data are representative of three independent experiments. One-way ANOVA was performed along with Bonferroni post test. *Asterisks* indicate statistically significant differences (*P* < 0.05) when compared with the luciferase activity level with 0 ng of AHR, AHRR expression vector transfection. *Double asterisks* and *hashtags* indicate statistically significant differences (*P* < 0.05) compared with the luciferase activity level to the absence of AHRR for DMSO and TCDD treatment, respectively. *n.s.* not significant

To further characterize AHR- and AHRR-specific regulation of the regions identified from our ChIP-Seq analysis we focused on the AHR-specific target gene, *CNR2* and the AHRR-specific target gene, *XRCC6*. The reporter plasmids and AHR, ARNT or AHRR were transfected into COS-1 cells, which express very low to negligible endogenous levels of AHR and ARNT (Long et al. 1999). *CYP1B1* reporter gene activity was increased after transfection with AHR and ARNT and further increased after TCDD treatment (Fig. 7a). As expected, transfection of AHRR repressed the TCDD-induced regulation of *CYP1B1* reporter gene activity (Fig. 7a). *CYP1B1* was classified as an AHR:AHRR-co-bound region from our ChIP-seq analysis, which is supported by the integrative genome viewer (IGV) visualization of the ChIP-Seq peak analysis for AHR and AHRR at *CYP1B1* (Fig. 7b). Similar to a previous report, only a small number of the identified AHREs were bound by AHR or AHRR across the *CYP1B1* upstream regulatory region (Dere et al. 2011). We next examined the unique AHR-bound region located within *CNR2* using a reporter gene assay (Fig. 7c). IGV visualization revealed specific recruitment of AHR to CNR2 (Fig. 7d). Because the AHR-bound region was located within *CNR2* and not near the promoter gene of the gene, we cloned this region into a pGL3-promoter vector to determine whether it could act as an AHR-regulated enhancer. Transfection of increasing amounts of AHR and ARNT resulted in an increase in TCDD-dependent reporter gene activity (Fig. 7c). However, overexpression of AHRR did not inhibit the AHR-dependent increases of *CNR2*-regulated reporter gene activity (Fig. 7c). Since the AHRR-bound ChIP region was located within the *XRCC6* promoter, we cloned approximately a 1-kb region of the *XRCC6* regulatory region that included the AHRR-bound ChIP region into pGL3-basic luciferase vector (Fig. 7e). IGV visualization showed specific recruitment of AHRR, but not AHR, to an AHRE dense region located upstream of the *XRCC6* TSS (Fig. 7f). The AHRR-bound region in *XRCC6* was also located in close proximity to the TSS of desumoylating isopeptidase 1 (DESI1); however, we focused on the promoter region of XRCC6 since it was the closest mapped gene. Overexpression of AHRR repressed *XRCC6*-regulated reporter gene activity in the absence of AHR and ARNT (Fig. 7e). Increasing amounts of AHR and ARNT did not affect nor rescue the AHRR-mediated repression of *XRCC6* reporter gene activity (Fig. 7e).

**Fig. 8:**
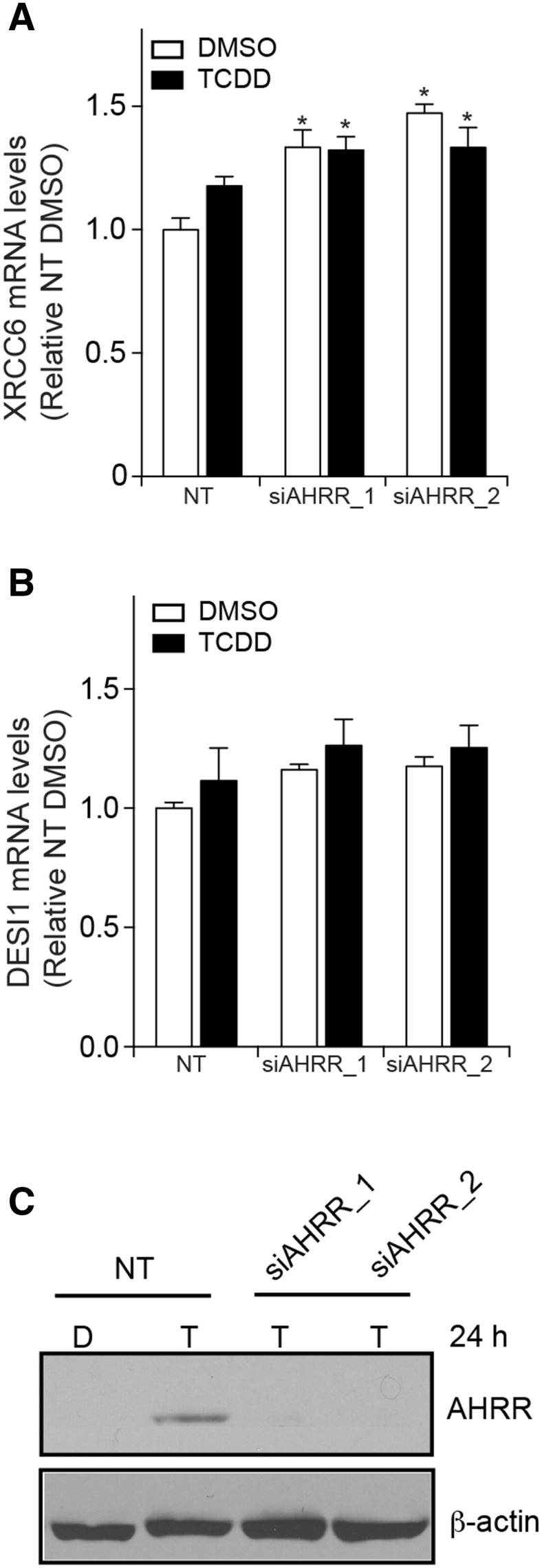
AHRR knockdown increases XRCC6 mRNA levels. **a** AHRR knockdown increases XRCC6 mRNA levels in MCF7 cells treated for 24 h with DMSO (D) or TCDD (T). **b** No significant changes in DESI1 mRNA levels were observed after AHRR knockdown in MCF7 cells treated with DMSO (D) or TCDD (T). RNAi-mediated knockdown of AHRR effectively reduced TCDD-dependent increases in AHRR protein levels. Data shown are representative of three independent experiments. *Asterisks* denoted significantly different (*P* < 0.05) gene expression differences compared with non-targeted (NT) DMSO-treated cells

We next determined the impact of AHRR knockdown on XRCC6 mRNA levels in MCF7 cells in the presence and absence of TCDD (Fig. 8a). Since *DESI1* gene was closely located to the AHRR-bound region that mapped to *XRCC6*, the effect of AHRR knockdown on DESI1 mRNAs was also examined (Fig. 8b). This was done to eliminate the possibility that the AHRR-bound region between *XRCC6* and *DESI1* functions as a bidirectional promoter regulating the expression of both genes. Because AHRR protein was not detected in DMSO-treated MCF-7 cells, the effectiveness of RNAi-mediated knockdown of AHRR protein levels was determined after 24-h TCDD treatment (Fig. 8c). In agreement with our previous report, significant reduction in AHRR protein levels after transfection of both siAHRR sequences compared with TCDD-treated non-targeting (NT) cells (Fig. 8c). Knockdown of AHRR resulted in a small, but significant increase in XRCC6 mRNA levels that was independent of TCDD (Fig. 8a). No significant changes in DESI1 mRNA levels were observed, suggesting that AHRR regulates XRCC6 and not DESI1 mRNA expression levels (Fig. 8b). These findings agreed with the gain of function studies shown in Fig. 7e where increased AHRR decreased XRCC6-regulated reporter gene activity, but its knockdown increased XRCC6 mRNA levels. CNR2 is preferentially expressed in the immune and nervous system (Anand et al. 2008; Galiegue et al. 1995), and not detected in MCF-7 cells. This suggests that AHR binding to *CNR2* might be a non-productive event in MCF-7 cells, or that AHR might regulate the expression of CNR2 in cell- or tissue-specific manner.

## Discussion

AHRR is well established as an AHR ligand-induced negative regulator of AHR activity; however, the molecular mechanisms, selectivity and whether AHRR functions as a general or gene-specific repressor of AHR are not well understood. AHRR represses AHR through binding to AHREs and by binding to ARNT (Mimura et al. 1999). Interestingly, overexpression of ARNT does not prevent AHRR-dependent repression of AHR, and DNA-binding mutant of AHRR is still able to repress AHR activity and AHRR interacts directly with AHR (Evans et al. 2008). These latest findings suggest that AHRR represses AHR signaling through direct interaction with AHR via direct binding to AHREs or through tethering to AHR or other transcription factors (Hahn et al. 2009). AHRR also represses hypoxia factor 1α (HIF1α) activity, but has limited effectiveness at repressing nuclear receptor-mediated transcription (Karchner et al. 2009). However, AHRR has been reported to repress estrogen receptor activity (Kanno et al. 2008). In an effort to better understand the AHR–AHRR signaling axis, we determined the genome-wide binding profiles of AHRR and AHR in TCDD-treated MCF-7 human breast cancer cells. Here we provide evidence that AHR and AHRR exhibit similar, but also distinct, binding profiles and that the AHRE is a prevalent motif in AHR:AHRR-co-bound regions, but also in AHR-only and AHRR-only regions.

The overall binding profiles of AHR were consistent with our previous work, but we identified more AHR-bound regions for both 45-min and 24-h datasets (Lo and Matthews 2012). The increased number of AHR-bound regions is most likely attributed to the different peak-calling algorithms and library preparation methods that we used (Lo and Matthews 2012). We found that AHR bound to only 34% of AHRR-bound regions or 44% of the closest genes after 24 h of TCDD treatment. However, approximately 65% of the AHRR-bound regions or 79% of the closest genes overlapped with those of AHR when we considered the three AHR_45min_, AHR_24h_, and AHRR_24h_ datasets. This supports the notion that AHR and AHRR may compete for or bind to the same target gene sequences (Evans et al. 2008; Mimura et al. 1999). Additional studies including re-ChIP experiments will be needed to determine if AHR and AHRR are present at these genomic sequences at the same time (Metivier et al. 2003). Moreover, it should be determined whether the genomic binding profiles of ARNT will be important to resolve its role in AHRR-dependent repression of AHR (Evans et al. 2008). The reduced overlap observed at 24 h may reflect temporal differences in recruitment of AHR and AHRR to the shared genomics regions, because many of the regions occupied by AHRR at 24 h were occupied by AHR at 45 min but not at 24 h. These findings imply that, in certain cases, AHRR may bind to regions recognized by AHR when AHR is not present. Studies of *Ahrr* ^−*/*−^ mice suggest that AHRR is a context and selective repressor of AHR (Hosoya et al. 2008), whereas other reports suggest that AHRR may regulate genes independent of AHR (Zudaire et al. 2008). This is supported by the AHRR-independent binding of AHR to *CNR2* and the AHR-independent regulation of *XRCC6* by AHRR. Determining the occupancy patterns of AHR and AHRR at multiple time points will be important future experiments to more accurately determine unique genomic regions bound by AHR and AHRR. There was also a higher degree of overlap between AHR and AHRR at annotated genes than at their corresponding genomic regions suggesting that in other cases AHR and AHRR may be binding to distinct regions of the same gene.

The major difference in the binding profiles between AHR and AHRR was that AHRR preferentially bound closer to promoter regions compared with the broader binding distribution of AHR. This suggests that AHRR selectively competes with or interacts with AHR at or close to promoter regions rather than at distal enhancer regions. As expected, de novo motif discovery and TFBS analyses identified the AHRE as a highly ranked motif in the AHR:AHRR-co-bound regions, AHR-only, and AHRR-only regions. Overall, the results obtained from DREME and SEME were comparable, except that SEME did not identify an AHRE-like sequence in the AHRR-only dataset. This was most likely due to the different algorithms used by the two programs. Forkhead box transcription factor motifs were identified in AHR:AHRR-co-bound, AHR-only, and AHRR-only regions (Ahmed et al. 2012). FOXA family of transcription factors together with GATA proteins are pioneer transcription factors and are necessary to mediate chromatin looping and facilitate transcriptional control (Zaret and Carroll 2011). FOXA1 and FOXA2 are essential transcription factors in cancer as they are able to bind to their response elements in tightly wrapped nucleosomes (Soufi et al. 2015). In breast cancer cells, FOXA1 is needed to reprogram the genomic binding profiles of estrogen receptor following ligand activation (Hurtado et al. 2011). FOXA1 is essential for TCDD-induced regulation of *CCNG2* by AHR (Ahmed et al. 2012). How the FOXA family contributes to AHRR signaling and AHRR-dependent repression of AHR is unknown. AHRR has also been proposed to function independently of AHR particularly in its role as a tumor suppressor (Kanno et al. 2008; Schlezinger et al. 2006). Interestingly, a number of binding sites for other tumor suppressors or cancer-related transcription factors, including E2F, EGR, KLF as well as STAT, were enriched or identified in regions uniquely bound by AHRR. The functional consequences of the presence of these transcription factor sites and whether AHRR interacts and/or functions together with these transcription factors remain to be determined.

Similar to AHRR, TIPARP, an AHR target gene and a mono-ADP-ribosyltransferase, also functions as part of a negative feedback loop to regulate AHR activity via mono-ADP-ribosylation (Ahmed et al. 2015; MacPherson et al. 2013). Although the sensitivity of *Ahrr* ^−*/*−^ mice to TCDD has not been reported, *Tiparp* ^−*/*−^ mice exhibit an increased sensitivity to TCDD toxicities and lethality (Ahmed et al. 2015). As with mRNA levels of other AHR target genes, including Cyp1a1 and Cyp1b1, Ahrr mRNA levels are more strongly induced after 6-h TCDD treatment of *Tiparp* ^−*/*−^ mice compared with wild-type mice (Ahmed et al. 2015). This might suggest that the increased sensitivity of the *Tiparp* ^−*/*−^ to TCDD is independent of the ability of AHRR to repress AHR. Although TIPARP catalytic activity is required to repress AHR and regulates ligand-induced proteolytic degradation of AHR, the molecular mechanisms are not well understood (Ahmed et al. 2015; MacPherson et al. 2013, 2014). For example, TIPARP mono-ADP-ribosylates AHR but not ARNT; however, whether AHRR or other transcription factors essential for AHR signaling are also post-translationally modified by TIPARP has not been determined. It will also be important to determine the contribution of both AHRR and TIPARP to the increasing important biological and immunological roles attributed to AHR (Stockinger et al. 2011).

One of the limitations of this study is that we treated the MCF-7 cells with TCDD for 24 h prior to doing the ChIP assays for AHRR. This was based on previous time course studies in which we did not observe high levels of AHRR in MCF-7 cells prior to TCDD treatment (MacPherson et al. 2014). Our studies were also done using a single antibody against AHRR, one cell line, and one time point. The single time point is important given the dynamic on and off binding of transcription factors to their response elements (Metivier et al. 2003). The AHRR- and AHR-bound regions were normalized to DMSO (solvent control) for AHRR and AHR, respectively, rather than compared to IgG or to total input chromatin. This provides a robust TCDD-dependent dataset for both transcription factors. Using a solo-peak-calling method we also gained information about the genomic binding patterns of AHR and AHRR in the absence of TCDD. Human cervix epithelial adenocarcinoma, HeLa cells have high endogenous mRNA levels of AHRR and could be used to determine the genomic binding profiles of AHRR without prior TCDD treatment (Tsuchiya et al. 2003).

In summary, this is the first study to report the combined genomic binding profiles of AHRR and AHR. Our findings reveal that AHR and AHRR exhibit similar, but also distinct, genome-wide binding profiles with AHRR preferentially binding to genomic sequences close to promoter regions. There was a relatively high degree of overlap between AHRR-bound and AHR-bound regions. These data support the view that, following TCDD treatment, AHRR is recruited to genomic regions occupied or previously occupied by AHR. However, the impact of AHRR recruitment to these regions (repression, activation or no effect) will require more detailed analyses. We also observed that, in some instances, AHRR and AHR bound to distinct genomic regions, suggesting that each transcription factor can function independently of the other. Moreover, the presence of AHREs and AHRE-like motifs in AHR-only and AHRR-only regions, suggests that there are AHREs and/or AHRE-like motifs that are differentially recognized by AHR and AHRR. The mechanisms and sequence characteristics that regulate the selective binding of AHR and AHRR to these motifs remain to be determined.

## Electronic supplementary material

Below is the link to the electronic supplementary material.

Supplementary material 1 (DOCX 18 kb)

Supplementary material 2 (DOCX 47 kb)

## References

[CR1] Ahmed S, Al-Saigh S, Matthews J (2012). FOXA1 is essential for aryl hydrocarbon receptor-dependent regulation of cyclin G2. Mol Cancer Res.

[CR2] Ahmed S, Bott D, Gomez A (2015). Loss of the mono-ADP-ribosyltransferase, TIPARP, increases sensitivity to dioxin-induced steatohepatitis and lethality. J Biol Chem.

[CR3] Anand U, Otto WR, Sanchez-Herrera D (2008). Cannabinoid receptor CB2 localisation and agonist-mediated inhibition of capsaicin responses in human sensory neurons. Pain.

[CR4] Bailey TL (2011). DREME: motif discovery in transcription factor ChIP-seq data. Bioinformatics.

[CR5] Consortium EP (2012). An integrated encyclopedia of DNA elements in the human genome. Nature.

[CR6] Dere E, Lo R, Celius T, Matthews J, Zacharewski TR (2011). Integration of genome-wide computation DRE search, AhR ChIP-chip and gene expression analyses of TCDD-elicited responses in the mouse liver. BMC Genom.

[CR7] Edgar R, Domrachev M, Lash AE (2002). Gene expression omnibus: NCBI gene expression and hybridization array data repository. Nucleic Acids Res.

[CR8] Evans BR, Karchner SI, Allan LL (2008). Repression of aryl hydrocarbon receptor (AHR) signaling by AHR repressor: role of DNA binding and competition for AHR nuclear translocator. Mol Pharmacol.

[CR9] Fernandez-Salguero P, Pineau T, Hilbert DM (1995). Immune system impairment and hepatic fibrosis in mice lacking the dioxin-binding Ah receptor. Science.

[CR10] Galiegue S, Mary S, Marchand J (1995). Expression of central and peripheral cannabinoid receptors in human immune tissues and leukocyte subpopulations. Eur J Biochem.

[CR11] Gu YZ, Hogenesch JB, Bradfield CA (2000). The PAS superfamily: sensors of environmental and developmental signals. Annu Rev Pharmacol Toxicol.

[CR12] Gualdrini F, Esnault C, Horswell S, Stewart A, Matthews N, Treisman R (2016). SRF co-factors control the balance between cell proliferation and contractility. Mol Cell.

[CR13] Hahn ME, Allan LL, Sherr DH (2009). Regulation of constitutive and inducible AHR signaling: complex interactions involving the AHR repressor. Biochem Pharmacol.

[CR14] Hankinson O (1995). The aryl hydrocarbon receptor complex. Annu Rev Pharmacol Toxicol.

[CR15] Heinz S, Benner C, Spann N (2010). Simple combinations of lineage-determining transcription factors prime cis-regulatory elements required for macrophage and B cell identities. Mol Cell.

[CR16] Hosoya T, Harada N, Mimura J (2008). Inducibility of cytochrome P450 1A1 and chemical carcinogenesis by benzo[a]pyrene in AhR repressor-deficient mice. Biochem Biophys Res Commun.

[CR17] Hurtado A, Holmes KA, Ross-Innes CS, Schmidt D, Carroll JS (2011). FOXA1 is a key determinant of estrogen receptor function and endocrine response. Nat Genet.

[CR18] Kanno Y, Takane Y, Takizawa Y, Inouye Y (2008). Suppressive effect of aryl hydrocarbon receptor repressor on transcriptional activity of estrogen receptor alpha by protein–protein interaction in stably and transiently expressing cell lines. Mol Cell Endocrinol.

[CR19] Karchner SI, Jenny MJ, Tarrant AM (2009). The active form of human aryl hydrocarbon receptor (AHR) repressor lacks exon 8, and its Pro 185 and Ala 185 variants repress both AHR and hypoxia-inducible factor. Mol Cell Biol.

[CR20] Kikuchi Y, Ohsawa S, Mimura J (2003). Heterodimers of bHLH-PAS protein fragments derived from AhR, AhRR, and Arnt prepared by co-expression in Escherichia coli: characterization of their DNA binding activity and preparation of a DNA complex. J Biochem (Tokyo).

[CR21] Langmead B, Salzberg SL (2012). Fast gapped-read alignment with Bowtie 2. Nat Methods.

[CR22] Li H, Handsaker B, Wysoker A (2009). The Sequence Alignment/Map format and SAMtools. Bioinformatics.

[CR23] Lo R, Matthews J (2012). High-resolution genome-wide mapping of AHR and ARNT binding sites by ChIP-Seq. Toxicol Sci.

[CR24] Lo R, Celius T, Forgacs A (2011). Identification of aryl hydrocarbon receptor binding targets in mouse hepatic tissue treated with 2,3,7,8-tetrachlorodibenzo-*p*-dioxin. Toxicol Appl Pharmacol.

[CR25] Long WP, Chen X, Perdew GH (1999). Protein kinase C modulates aryl hydrocarbon receptor nuclear translocator protein-mediated transactivation potential in a dimer context. J Biol Chem.

[CR26] Ma Q, Baldwin KT, Renzelli AJ, McDaniel A, Dong L (2001). TCDD-inducible poly(ADP-ribose) polymerase: a novel response to 2,3,7,8-tetrachlorodibenzo-*p*-dioxin. Biochem Biophys Res Commun.

[CR27] MacPherson L, Lo R, Ahmed S, Pansoy A, Matthews J (2009). Activation function 2 mediates dioxin-induced recruitment of estrogen receptor alpha to CYP1A1 and CYP1B1. Biochem Biophys Res Commun.

[CR28] MacPherson L, Tamblyn L, Rajendra S, Bralha F, McPherson JP, Matthews J (2013). 2,3,7,8-tetrachlorodibenzo-*p*-dioxin poly(ADP-ribose) polymerase (TiPARP, ARTD14) is a mono-ADP-ribosyltransferase and repressor of aryl hydrocarbon receptor transactivation. Nucleic Acids Res.

[CR29] MacPherson L, Ahmed S, Tamblyn L (2014). Aryl hydrocarbon receptor repressor and TiPARP (ARTD14) use similar, but also distinct mechanisms to repress aryl hydrocarbon receptor signaling. Int J Mol Sci.

[CR30] Mahony S, Benos PV (2007). STAMP: a web tool for exploring DNA-binding motif similarities. Nucleic Acids Res.

[CR31] Metivier R, Penot G, Hubner MR (2003). Estrogen receptor-alpha directs ordered, cyclical, and combinatorial recruitment of cofactors on a natural target promoter. Cell.

[CR32] Mimura J, Ema M, Sogawa K, Fujii-Kuriyama Y (1999). Identification of a novel mechanism of regulation of Ah (dioxin) receptor function. Genes Dev.

[CR33] Puga A, Xia Y, Elferink C (2002). Role of the aryl hydrocarbon receptor in cell cycle regulation. Chem Biol Interact.

[CR34] Quinlan AR, Hall IM (2010). BEDTools: a flexible suite of utilities for comparing genomic features. Bioinformatics.

[CR35] Rannug A, Rannug U, Rosenkranz HS (1987). Certain photooxidized derivatives of tryptophan bind with very high affinity to the Ah receptor and are likely to be endogenous signal substances. J Biol Chem.

[CR36] Schlezinger JJ, Liu D, Farago M (2006). A role for the aryl hydrocarbon receptor in mammary gland tumorigenesis. Biol Chem.

[CR37] Schmidt JV, Su GH, Reddy JK, Simon MC, Bradfield CA (1996). Characterization of a murine Ahr null allele: involvement of the Ah receptor in hepatic growth and development. Proc Natl Acad Sci USA.

[CR38] Soufi A, Garcia MF, Jaroszewicz A, Osman N, Pellegrini M, Zaret KS (2015). Pioneer transcription factors target partial DNA motifs on nucleosomes to initiate reprogramming. Cell.

[CR39] Stockinger B, Hirota K, Duarte J, Veldhoen M (2011). External influences on the immune system via activation of the aryl hydrocarbon receptor. Semin Immunol.

[CR40] Thorvaldsdottir H, Robinson JT, Mesirov JP (2013). Integrative Genomics Viewer (IGV): high-performance genomics data visualization and exploration. Brief Bioinform.

[CR41] Tsuchiya Y, Nakajima M, Itoh S, Iwanari M, Yokoi T (2003). Expression of aryl hydrocarbon receptor repressor in normal human tissues and inducibility by polycyclic aromatic hydrocarbons in human tumor-derived cell lines. Toxicol Sci.

[CR42] Zaret KS, Carroll JS (2011). Pioneer transcription factors: establishing competence for gene expression. Genes Dev.

[CR43] Zhang Y, Liu T, Meyer CA (2008). Model-based analysis of ChIP-Seq (MACS). Genome Biol.

[CR44] Zhang Z, Chang CW, Hugo W, Cheung E, Sung WK (2013). Simultaneously learning DNA motif along with its position and sequence rank preferences through expectation maximization algorithm. J Comput Biol.

[CR45] Zudaire E, Cuesta N, Murty V (2008). The aryl hydrocarbon receptor repressor is a putative tumor suppressor gene in multiple human cancers. J Clin Invest.

