# Supplementary material for: Genome-wide mapping and analysis of aryl hydrocarbon receptor (AHR)- and aryl hydrocarbon receptor repressor (AHRR)-binding sites in human breast cancer cells
Source: Arch Toxicol. 2017 Jul 5;92(1):225–40. doi: 10.1007/s00204-017-2022-x (PMC5773648; doi:10.1007/s00204-017-2022-x)
Supplement: Supplementary file 1 — Supplementary material 1 (DOCX 18 kb) [file 204_2017_2022_MOESM1_ESM.docx]

| Table S1. Q-PCR primers used for the ChIP-qPCR validation of selected unique binding regions extracted from ChIP-seq analysis | | | | | |
| --- | --- | --- | --- | --- | --- |
| Chrom | Start | End | Closest Gene | Dataset | Primer sequence 5’-3’ |
| Chr6 | 112497602 | 112497711 | LAMA4 | AHR-only | *For-*TGGTTGACCAGACAAGATGGG |
|  |  |  |  |  | *Rev-*AAATAACCGCTGGCGACCTT |
| Chr4 | 185725042 | 185725147 | ACSL1 | AHR-only | *For-*CGTCTCTGCTCTCGTCCATC |
|  |  |  |  |  | *Rev-*CCCTTAGGTGGTTTGGGCTT |
| Chr3 | 16551179 | 16551271 | *RFTN1* | AHR-only | *For-*AGCCATGGTAAAGTGCCCTC |
|  |  |  |  |  | *Rev-*CATCTGGGTGATCGCCTCTC |
| Chr1 | 24223771 | 24223848 | *CNR2* | AHR-only | *For-*GGCGAGAGAAAGGCAGATCA |
|  |  |  |  |  | *Rev-*AAGACGGTAAAGGGTCGCTG |
| Chr19 | 7968539 | 7968637 | *MAP2K7* | AHRR-only | *For-*TCAGGAGCGATCGGGAATTG |
|  |  |  |  |  | *Rev-*GATGACGCCACCTAGAGCTC |
| Chr17 | 46018732 | 46018826 | *PNPO* | AHRR-only | *For-*GCGGTGACAGCATTTCCTTG |
|  |  |  |  |  | *Rev-*ACGAGGCCAATTAGAGCGAG |
| Chr1 | 6259664 | 6259734 | *RPL22* | AHRR-only | *For-*CGAACTACGCAGACGCAAAG |
|  |  |  |  |  | *Rev-*GCAAAGTGATTAGTGCGGCC |
| Chr22 | 42017193 | 42017286 | *XRCC6* | AHRR-only | *For-*AGGTAGAAGCTGGTTGGGGA |
|  |  |  |  |  | *Rev-*TACTCGGTCCCAATCAACGC |
