# Supplementary material for: Genome-wide mapping and analysis of aryl hydrocarbon receptor (AHR)- and aryl hydrocarbon receptor repressor (AHRR)-binding sites in human breast cancer cells
Source: Arch Toxicol. 2017 Jul 5;92(1):225–40. doi: 10.1007/s00204-017-2022-x (PMC5773648; doi:10.1007/s00204-017-2022-x)
Supplement: Supplementary file 2 — Supplementary material 2 (DOCX 47 kb) [file 204_2017_2022_MOESM2_ESM.docx]

| Table S2. Primers used for cloning and building of luciferase construct. | | | | | | |
| --- | --- | --- | --- | --- | --- | --- |
| Chrom | Start | End | Closest Gene | Dataset Source | Luciferase construct | Primer sequence 5’-3’ |
|  |  |  |  |  |  |  |
| Chr22 | 42016612 | 42017621 | *XRCC6* | AHRR-only | pGL-Basic | *For-*CAAAACGCGTCATGTGCTTACAGTCCTGACGT |
|  |  |  |  |  |  | *Rev-*CAAAAGATCTCAACACAAATGGATAACGGCCC |
| Chr1 | 24223513 | 24223945 | *CNR2* | AHR-only | pGL-Promoter | *For-*CAAAACGCGTAAGTTCTCAGTTTTAGTCTCTA |
|  |  |  |  |  |  | *Rev-*CAAAAGATCTTGGGGTTTCACTATTTTGGCCA |
